# Supplementary material for: Cardiological Reference Intervals in Adult American Staffordshire Terrier Dogs
Source: Animals (Basel). 2023 Jul 27;13(15):2436. doi: 10.3390/ani13152436 (PMC10417027; doi:10.3390/ani13152436)
Supplement: Supplementary file 1 [file animals-13-02436-s001.zip › animals-2447561-supplementary.pdf]

Table S1. Descriptive statistics of cardiological measurements obtained in a study population of 29 healthy adult American Staffordshire Terriers.

Cardiological measurement	Arithmetic mean (standard deviation)	Median (interquartile range)	Range	Test of normality p-value	Skewness coefficient (CI 95%)	Kurtosis coefficient (CI 95%)
SBP [mmHg]	133 (23)	137 (120 – 143)	80 – 180	0.972	-0.19 (-1.08 – 0.70)	0.02 (-1.71 – 1.76)
VHS	10.9 (0.6)	11.0 (10.5 – 11.4)	9.9 – 12.2	0.663	0.31 (-0.58 – 1.20)	-0.49 (-2.22 – 1.24)
**Electrocardiography**						
HR [min^{-1}]	109 (19)	110 (100 – 120)	70 – 150	0.322	0.17 (-0.72 – 1.06)	-0.02 (-1.75 – 1.71)
P [s]	0.05 (0.01)	0.05 (0.04 – 0.05)	0.03 – 0.06	0.002	-0.25 (-1.13 – 0.64)	0.04 (-1.69 – 1.77)
P [mV]	0.28 (0.08)	0.25 (0.25 – 0.30)	0.15 – 0.45	0.019	0.59 (-0.30 – 1.48)	0.36 (-1.37 – 2.09)
PQ [s]	0.11 (0.02)	0.10 (0.10 – 0.12)	0.07 – 0.14	0.348	0.07 (-0.81 – 0.96)	0.14 (-1.60 – 1.87)
QRS [s]	0.07 (0.01)	0.07 (0.06 – 0.08)	0.04 – 0.08	0.001	-1.17 (-2.06 – -0.28)*L	2.39 (0.66 – 4.13)*LK
Q [mV]	0.48 (0.19)	0.45 (0.35 – 0.65)	0.20 – 0.90	0.205	0.43 (-0.46 – 1.32)	-0.62 (-2.35 – 1.11)
R [mV]	1.80 (0.55)	1.80 (1.40 – 2.05)	0.90 – 3.20	0.279	0.69 (-0.20 – 1.58)	0.38 (-1.35 – 2.11)
S [mV]	0.18 (0.14)	0.15 (0.10 – 0.20)	0.03 – 0.60	0.000	1.78 (0.89 – 2.67)*R	2.83 (1.10 – 4.57)*LK
QT [s]	0.21 (0.02)	0.21 (0.20 – 0.22)	0.15 – 0.25	0.048	-0.55 (-1.44 – 0.33)	2.11 (0.38 – 3.85)*LK
ST [mV]	-0.14 (0.11)	-0.15 (-0.20 – -0.10)	-0.35 – 0.25	0.016	1.26 (0.37 – 2.14)*R	3.71 (1.97 – 5.44)*LK
T [mV]	0.19 (0.09)	0.15 (0.15 – 0.25)	0.01 – 0.35	0.132	0.27 (-0.62 – 1.16)	-0.54 (-2.27 – 1.19)
Mean electrical axis (MEA)	31 (20)	30 (15 – 46)	-10 – 62	0.311	-0.24 (-1.13 – 0.64)	-0.71 (-2.45 – 1.02)
**Echocardiography**						
RV [cm]	1.25 (0.32)	1.30 (1.00 – 1.40)	0.60 – 1.90	0.833	0.18 (-0.7 – 1.07)	-0.43 (-2.16 – 1.30)
IVSd [cm]	1.06 (0.14)	1.05 (1.00 – 1.17)	0.80 – 1.40	0.587	0.33 (-0.56 – 1.22)	-0.04 (-1.77 – 1.69)
IVSs [cm]	1.41 (0.20)	1.40 (1.21 – 1.60)	1.10 – 1.80	0.134	0.29 (-0.6 – 1.17)	-0.88 (-2.61 – 0.85)
LVDd [cm]	4.42 (0.34)	4.40 (4.10 – 4.60)	3.70 – 5.30	0.804	0.24 (-0.64 – 1.13)	0.33 (-1.40 – 2.06)
LVDs [cm]	3.17 (0.29)	3.17 (3.00 – 3.38)	2.49 – 3.70	0.888	-0.31 (-1.2 – 0.58)	-0.11 (-1.85 – 1.62)
LVWd [cm]	0.94 (0.12)	0.92 (0.89 – 1.00)	0.80 – 1.30	0.010	1.11 (0.22 – 2.00)*R	1.96 (0.23 – 3.70)*LK
LVWs [cm]	1.28 (0.20)	1.21 (1.20 – 1.40)	0.92 – 1.80	0.083	0.77 (-0.12 – 1.66)	0.94 (-0.79 – 2.67)
FS [%]	28.3 (4.6)	28.0 (26.0 – 31.0)	19.0 – 38.0	0.604	0.39 (-0.50 – 1.27)	0.15 (-1.59 – 1.88)
EF [%]	54.5 (6.7)	54.0 (51.0 – 59.0)	40.0 – 68.0	0.767	0.16 (-0.72 – 1.05)	0.03 (-1.70 – 1.76)
Ao [cm]	2.21 (0.17)	2.2 (2.10 – 2.36)	1.90 – 2.60	0.284	0.11 (-0.77 – 1.00)	-0.42 (-2.15 – 1.32)
LA [cm]	2.77 (0.31)	2.7 (2.56 – 2.98)	2.20 – 3.40	0.553	0.34 (-0.55 – 1.22)	-0.39 (-2.12 – 1.34)
LA/Ao	1.26 (0.12)	1.26 (1.16 – 1.35)	1.01 – 1.49	0.800	-0.19 (-1.08 – 0.69)	-0.63 (-2.36 – 1.10)
EPSS [cm]	0.53 (0.14)	0.50 (0.40 – 0.68)	0.30 – 0.80	0.062	0.22 (-0.66 – 1.11)	-1.18 (-2.91 – 0.55)

RVOT [m/s]	0.96 (0.21)	0.98 (0.77 – 1.12)	0.58 – 1.31	0.220	-0.14 (-1.03 – 0.75)	-1.16 (-2.90 – 0.57)
RVOT [mmHg]	3.84 (1.62)	3.8 (2.4 – 5.0)	1.30 – 6.80	0.167	0.13 (-0.76 – 1.02)	-1.19 (-2.92 – 0.54)
LVOT [m/s]	1.34 (0.30)	1.30 (1.13 – 1.44)	0.92 – 2.20	0.004	1.38 (0.49 – 2.27)*R	2.43 (0.70 – 4.17)*LK
LVOT [mmHg]	7.45 (3.60)	6.80 (5.10 – 8.30)	3.40 – 19.40	0.000	1.97 (1.09 – 2.86)*R	4.61 (2.88 – 6.34)*LK
Mitral valve E wave velocity [m/s]	0.79 (0.17)	0.77 (0.67 – 0.9)	0.52 – 1.14	0.551	0.30 (-0.59 – 1.19)	-0.65 (-2.38 – 1.09)
Mitral valve A wave velocity [m/s]	0.66 (0.14)	0.65 (0.58 – 0.78)	0.41 – 0.99	0.690	0.36 (-0.53 – 1.25)	-0.33 (-2.06 – 1.40)
E/A	1.22 (0.20)	1.20 (1.12 – 1.32)	0.82 – 1.71	0.537	0.34 (-0.55 – 1.23)	0.55 (-1.18 – 2.28)
EDV PLAX	65.8 (14.1)	64.0 (57.2 – 73.0)	42.0 – 104.0	0.456	0.71 (-0.18 – 1.60)	0.82 (-0.91 – 2.55)
ESV PLAX	32.0 (8.6)	33.0 (26.0 – 36.0)	15.0 – 51.0	0.531	0.45 (-0.44 – 1.34)	0.18 (-1.56 – 1.91)
EDV left A4C	61.1 (14.2)	60.5 (53.8 – 67.0)	38.0 – 91.0	0.579	0.26 (-0.63 – 1.15)	-0.51 (-2.25 – 1.22)
ESV left A4C	31.7 (9.9)	30.4 (23.3 – 35.0)	21.0 – 65.0	0.001	1.63 (0.74 – 2.52)*R	3.47 (1.74 – 5.20)*LK
LVDdN	1.63 (0.11)	1.63 (1.54 – 1.72)	1.46 – 1.83	0.228	0.32 (-0.56 – 1.21)	-0.93 (-2.66 – 0.80)
LVDsN	1.09 (0.10)	1.09 (1.03 – 1.15)	0.89 – 1.29	0.923	-0.03 (-0.92 – 0.85)	-0.19 (-1.92 – 1.54)
EDVI PLAX [ml/m^2]	67.5 (12.7)	66.4 (62.4 – 75.9)	45.2 – 91.9	0.400	0.04 (-0.85 – 0.93)	-0.51 (-2.24 – 1.23)
ESVI PLAX [ml/m^2]	32.7 (7.9)	33.9 (26.0 – 37.1)	18.1 – 52.3	0.551	0.30 (-0.58 – 1.19)	0.15 (-1.58 – 1.88)
EDVI left A4C [ml/m^2]	63.0 (14.3)	63.6 (53.9 – 73.8)	39.0 – 94.0	0.358	-0.04 (-0.93 – 0.85)	-0.63 (-2.36 – 1.10)
ESVI left A4C [ml/m^2]	32.4 (9.0)	30.2 (26.5 – 36.1)	20.2 – 60.2	0.008	1.38 (0.49 – 2.27)*R	2.31 (0.58 – 4.04)*LK

* significant deviation from symmetric or normal shape of distribution: R – right-hand asymmetry, L – left-hand asymmetry, LK – leptokurtic shape of distribution

Table S2. Relationship between cardiological measurements and demographic characteristics of dogs (gender, age, and body weight).

Cardiological measurement	Gender [a]			Age		Body weight	
	Males (n=15)	Females (n=14)	p-value	Correlation coefficient [b]	p-value	Correlation coefficient [b]	p-value
SBP [mmHg]	136 (19)	129 (26)	0.407	0.00	0.998	-0.13	0.504
VHS	11.0 (0.5)	10.9 (0.7)	0.871	-0.16	0.413	0.12	0.519
**Electrocardiography**							
HR [min^{-1}]	110 (24)	109 (13)	0.844	0.01	0.948	-0.16	0.412
P [s] [c]	0.05 (0.04 – 0.05)	0.05 (0.04 – 0.05)	0.810	0.06	0.764	-0.01	0.971
P [mV] [c]	0.25 (0.25 – 0.30)	0.28 (0.20 – 0.35)	0.983	0.12	0.529	0.03	0.887
PQ [s]	0.11 (0.02)	0.11 (0.02)	0.917	-0.11	0.572	0.14	0.470
QRS [s] [c]	0.06 (0.06 – 0.07)	0.07 (0.07 – 0.08)	0.002*	0.09	0.653	-0.05	0.801
Q [mV]	0.48 (0.23)	0.48 (0.15)	0.921	-0.47	0.010*	-0.09	0.659

R [mV]	2.03 (0.55)	1.54 (0.44)	0.014*	-0.22	0.261	0.04	0.835
S [mV] [c]	0.18 (0.10 – 0.35)	0.11 (0.10 – 0.15)	0.106	0.29	0.121	0.09	0.648
QT [s] [c]	0.20 (0.20 – 0.22)	0.21 (0.20 – 0.22)	0.600	-0.12	0.526	-0.37	0.046*
ST [mV] [c]	-0.15 (-0.20 – -0.10)	-0.15 (-0.20 – -0.10)	0.965	0.06	0.760	-0.23	0.231
T [mV]	0.23 (0.09)	0.16 (0.07)	0.039*	-0.12	0.529	-0.24	0.207
Mean electrical axis (MEA)	33 (18)	28 (23)	0.534	0.13	0.490	-0.13	0.487
**Echocardiography**							
RV [cm]	1.29 (0.33)	1.21 (0.31)	0.513	0.31	0.102	-0.07	0.710
IVSd [cm]	1.11 (0.15)	1.01 (0.12)	0.065	0.23	0.229	0.49	0.007*
IVSs [cm]	1.41 (0.23)	1.41 (0.18)	0.984	-0.25	0.187	0.36	0.055
LVDd [cm]	4.42 (0.41)	4.42 (0.26)	0.994	0.17	0.389	0.55	0.002*
LVDs [cm]	3.15 (0.33)	3.21 (0.24)	0.588	0.40	0.033*	0.33	0.080
LVWd [cm]	0.94 (0.90 – 1.02)	0.90 (0.80 – 1.00)	0.077	0.03	0.887	0.30	0.109
LVWs [cm]	1.31 (0.17)	1.26 (0.22)	0.490	-0.20	0.304	0.21	0.269
FS [%]	29.2 (3.8)	27.4 (5.2)	0.305	-0.33	0.085	0.20	0.302
EF [%]	55.9 (5.6)	53.1 (7.8)	0.272	-0.33	0.081	0.18	0.357
Ao [cm]	2.22 (0.12)	2.21 (0.21)	0.866	-0.17	0.381	0.06	0.762
LA [cm]	2.73 (0.24)	2.81 (0.37)	0.492	-0.39	0.037*	-0.03	0.891
LA/Ao	1.23 (0.11)	1.28 (0.14)	0.279	-0.33	0.080	-0.09	0.640
EPSS [cm]	0.54 (0.15)	0.52 (0.14)	0.728	-0.20	0.306	-0.01	0.943
RVOT [m/s]	1.03 (0.21)	0.88 (0.19)	0.059	-0.61	0.001*	0.10	0.611
RVOT [mmHg]	4.41 (1.64)	3.24 (1.43)	0.052	-0.58	0.001*	0.08	0.673
LVOT [m/s]	1.35 (1.17 – 1.52)	1.22 (1.04 – 1.41)	0.239	-0.18	0.362	-0.23	0.221
LVOT [mmHg]	7.3 (5.5 – 9.2)	5.95 (4.3 – 7.9)	0.239	-0.18	0.362	-0.23	0.221
Mitral valve E wave velocity [m/s]	0.82 (0.14)	0.76 (0.19)	0.328	-0.36	0.057	-0.05	0.788
Mitral valve A wave velocity [m/s]	0.70 (0.14)	0.63 (0.15)	0.197	-0.06	0.754	0.02	0.927
E/A	1.21 (0.22)	1.23 (0.20)	0.765	-0.34	0.074	-0.14	0.458
EDV PLAX	67.2 (16.1)	64.2 (12.1)	0.579	0.08	0.668	0.40	0.033*
ESV PLAX	33.7 (9.8)	30.1 (7.1)	0.268	0.38	0.041*	0.41	0.028*
EDV left A4C	63.5 (14.9)	58.5 (13.4)	0.351	0.00	0.990	0.20	0.304
ESV left A4C [c]	33.0 (23.3 – 38.0)	27.6 (23.0 – 34.2)	0.295	0.18	0.360	0.35	0.061
LVDdN	1.61 (0.11)	1.64 (0.10)	0.420	0.15	0.438	-0.01	0.968
LVDsN	1.07 (0.09)	1.11 (0.10)	0.220	0.37	0.049*	-0.19	0.335
EDVI PLAX [ml/m^2]	67.7 (14.5)	67.33 (11.1)	0.937	0.04	0.834	-0.06	0.771
ESVI PLAX [ml/m^2]	33.8 (8.8)	31.5 (7.0)	0.446	0.39	0.038*	0.09	0.651
EDVI left A4C [ml/m^2]	64.4 (15.4)	61.5 (13.5)	0.599	-0.04	0.845	-0.22	0.254
ESVI left A4C [ml/m^2] [c]	30.2 (27.0 – 41.9)	30.0 (25.6 – 35.9)	0.527	0.14	0.457	0.04	0.823

[a] numerical values presented as the arithmetic mean and standard deviation except for non-normally distributed presented as the median and interquartile range
[b] assessed using the Pearson's product-moment correlation coefficient (R) except for non-normally distributed assessed using the Spearman's rank correlation coefficient (R_s)
[c] non-normally distributed according to the Shapiro-Wilk test
* significant at α=0.05

Table S3. Reference intervals (RI) for cardiological measurements obtained in a study population of 29 healthy adult American Staffordshire Terriers and commonly accepted cariological RIs.

Cardiological measurement	Lower RI limit (CI 90%)	Upper RI limit (CI 90%)	Method [a]	Commonly used RI	Reference	RI specific for American Staffordshire Terriers (Vezzosi et al. 2021)
SBP [mmHg]	85 (74 – 97)	180 (168 – 192)	UTS	<150	[41]	ND
VHS	9.8 (9.5 – 10.1)	12.1 (11.8 – 12.4)	BCTS	8.5 – 10.6	[3]	ND
**Electrocardiography**						
HR [min^{-1}]	70 (59 – 80)	149 (138 – 159)	UTS	70 – 160	[10]	ND
P [s]	0.03 (0.02 – 0.04)	0.06 (0.05 – 0.07)	BCTS	<0.05	[10]	ND
P [mV]	0.14 (0.11 – 0.17)	0.46 (0.40 – 0.52)	BCTS	≤0.4	[10]	ND
PQ [s]	0.07 (0.07 – 0.08)	0.14 (0.13 – 0.15)	UTS	0.06 – 0.13	[10]	ND
QRS [s]	0.04 (0.03 – 0.05)	0.08 (0.08 – 0.09)	BCTS	≤0.06	[10]	ND
Q [mV]	0.16 (0.11 – 0.22)	0.94 (0.79 – 1.09)	UTS	ND	ND	ND
R [mV]	0.88 (0.72 – 1.04)	3.15 (2.70 – 3.68)	BCTS	≤3.0	[10]	ND
S [mV]	0.03 (0.02 – 0.05)	0.66 (0.40 – 1.05)	BCTS	ND	ND	ND
QT [s]	0.16 (0.15 – 0.18)	0.25 (0.24 – 0.26)	BCTS	0.15 – 0.25	[10]	ND
ST [mV]	-0.33 (-0.36 – -0.29)	0.14 (0.04 – 0.26)	BCTS	-0.2 – 0.15	[10]	ND
T [mV]	0.01 (-0.04 – 0.06)	0.38 (0.32 – 0.42)	UTS	0.05 – 1.00	[10]	ND
Mean electrical axis (MEA)	-10.9 (-21.5 – 0.30)	72.3 (60.4 – 83.1)	UTS	40 – 100	[10]	ND
**Echocardiography**						
RV [cm]	0.58 (0.40 – 0.76)	1.91 (1.74 – 2.08)	UTS	ND	ND	ND
IVSd [cm]	0.76 (0.68 – 0.84)	1.36 (1.28 – 1.44)	UTS	1.02 – 1.13	[7]	0.59 – 1.43
IVSs [cm]	0.98 (0.87 – 1.09)	1.83 (1.74 – 1.95)	UTS	1.54 – 1.70	[7]	0.81 – 2.16
LVDd [cm]	3.70 (3.50 – 3.87)	5.13 (4.93 – 5.32)	UTS	3.70 – 3.84	[7]	3.44 – 5.12
LVDs [cm]	2.57 (2.42 – 2.73)	3.78 (3.60 – 3.93)	UTS	2.32 – 2.44	[7]	1.76 – 3.69
LVWd [cm]	0.76 (0.73 – 0.80)	1.25 (1.13 – 1.38)	BCTS	0.82 – 0.91	[7]	0.62 – 1.21

LVWs [cm]	0.95 (0.89 – 1.02)	1.78 (1.59 – 1.97)	BCTS	1.32 – 1.44	[7]	0.93 – 1.91
FS [%]	18.8 (16.5 – 21.3)	37.8 (35.2 – 40.1)	UTS	25 – 39	[10]	20.1 – 56.4
EF [%]	40.5 (36.6 – 44.0)	68.6 (64.7 – 72.3)	UTS	50 – 65	[10]	40.5 – 82.5
Ao [cm]	1.86 (1.77 – 1.94)	2.56 (2.47 – 2.65)	UTS	2.53 – 2.70	[7]	1.39 – 2.37
LA [cm]	2.14 (1.99 – 2.31)	3.41 (3.24 – 3.57)	UTS	2.39 – 2.59	[7]	ND
LA/Ao	1.00 (0.94 – 1.07)	1.51 (1.44 – 1.57)	UTS	1.00 – 1.68	[54]	1.00 – 1.75
EPSS [cm]	0.28 (0.24 – 0.33)	0.89 (0.76 – 1.02)	BCTS	0.5 – 0.8	[10]	ND
RVOT [m/s]	0.51 (0.39 – 0.63)	1.40 (1.28 – 1.52)	UTS	<1.5	[10]	0.76 – 1.70
RVOT [mmHg]	0.99 (0.56 – 1.57)	7.77 (6.56 – 9.12)	BCTS	ND	ND	ND
LVOT [m/s]	0.93 (0.88 – 1.00)	2.14 (1.81 – 2.51)	BCTS	<1.7	[10]	1.17 – 2.45
LVOT [mmHg]	3.4 [b]	14.9 (10.6 – 18.1)	BCTS	ND	ND	ND
Mitral valve E wave velocity [m/s]	0.45 (0.35 – 0.54)	1.14 (1.04 – 1.23)	UTS	0.58 – 1.17	[55]	0.50 – 1.29
Mitral valve A wave velocity [m/s]	0.36 (0.28 – 0.44)	0.96 (0.89 – 1.04)	UTS	0.39 – 0.86	[55]	0.36 – 1.16
E/A	0.79 (0.68 – 0.92)	1.64 (1.52 – 1.75)	UTS	0.92 – 2.72	[55]	0.90 – 1.88
EDV PLAX	41.8 (37.8 – 47.0)	100.4 (88.7 – 112.5)	BCTS	41.1 – 101.6	[34]	ND
ESV PLAX	13.9 (9.5 – 18.9)	50.0 (45.3 – 54.5)	UTS	18.4 – 45.6	[34]	ND
EDV left A4C	31.6 (24.4 – 39.7)	90.6 (81.9 – 98.5)	UTS	41.1 – 101.6	[34]	ND
ESV left A4C	19.4 (17.7 – 21.0)	65.1 (49.2 – 103.2)	BCTS	18.4 – 45.6	[34]	ND
LVDdN	1.41 (1.35 – 1.47)	1.85 (1.80 – 1.91)	UTS	<1.85	[8]	1.27 – 1.87
LVDsN	0.89 (0.83 – 0.95)	1.29 (1.24 – 1.34)	UTS	<1.26	[8]	0.64 – 1.31
EDVI PLAX [ml/m^2]	41.0 (33.8 – 48.8)	94.1 (86.5 – 101.1)	UTS	ND	ND	37.1 – 86.7
ESVI PLAX [ml/m^2]	16.2 (12.0 – 20.8)	49.2 (44.6 – 53.4)	UTS	ND	ND	9.5 – 35.3
EDVI left A4C [ml/m^2]	33.1 (25.3 – 41.0)	92.8 (84.6 –101.1)	UTS	ND	ND	ND
ESVI left A4C [ml/m^2]	20.2 [b]	50.9 (41.3 – 56.9)	BCTS	ND	ND	ND

[a] UTS – untransformed standard method, BCTS – Box-Cox transformed standard method
[b] replaced with the lowest observed value if the CI 90% included 0.

ND – not determined
